# Tolerance testing of passive radio frequency identification tags for solvent, temperature, and pressure conditions encountered in an anatomic pathology or biorepository setting

**DOI:** 10.4103/2153-3539.70710

**Published:** 2010-10-01

**Authors:** Alina A. Leung, Jerry J. Lou, Sergey Mareninov, Steven S. Silver, Mark J. Routbort, Michael Riben, Gary Andrechak, William H. Yong

**Affiliations:** 1 Department of Pathology and Laboratory Medicine (Neuropathology), David Geffen School of Medicine at UCLA, Los Angeles, CA; 2 Jonsson Cancer Center, David Geffen School of Medicine at UCLA, Los Angeles, CA; 3 Brain Research Institute, David Geffen School of Medicine at UCLA, Los Angeles, CA; 4 Department of Pathology, University of Texas MD Anderson Cancer Center, Houston, TX; 5 Department Hematopathology, University of Texas MD Anderson Cancer Center, Houston, TX; 6 New Age Industries / AdvantaPure, Southampton, PA

**Keywords:** Autoclave, radio frequency identification tags, RFID, solvent, temperature

## Abstract

**Background:**

Radio frequency identification (RFID) tags have potential for use in identifying and tracking biospecimens in anatomic pathology and biorepository laboratories. However, there is little to no data on the tolerance of tags to solutions, solvents, temperatures, and pressures likely to be encountered in the laboratory. The functioning of the Hitachi Mu-chip RFID tag, a candidate for pathology use, was evaluated under such conditions.

**Methods:**

The RFID tags were affixed to cryovials containing tissue or media, glass slides, and tissue cassettes. The tags were interrogated for readability before and after each testing condition or cycle. Individual tags were subjected to only one testing condition but for multiple cycles. Testing conditions were: 1) Ten wet autoclave cycles (121°C, 15 psi); 2) Ten dry autoclave cycles (121°C, 26 psi); 3) Ten tissue processor cycles; 4) Ten hematoxylin and eosin (H&E) staining cycles; 5) Ten antigen retrieval pressure cooker cycles (125°C, 15 psi); 6) 75°C for seven days; 7) 75-59 °C day/night cycles for 7 days; 8) -80°C, -150°C, or -196°C for 12 months; 9) Fifty freeze-thaw cycles (-196°C to 22°C).

**Results:**

One hundred percent of tags exposed to cold temperatures from -80 to -196 °C (80 tags, 1120 successful reads), high temperatures from 52 to 75°C (40 tags, 420 reads), H & E staining (20 tags, 200 reads), pressure cooker antigen retrieval (20 tags, 200 reads), and wet autoclaving (20 tags, 200 reads) functioned well throughout and after testing. Of note, all 20 tested tags tolerated 50 freeze-thaw cycles and all 60 tags subjected to sustained freezing temperatures were readable after 1 year. One dry autoclaved tag survived nine cycles but failed after the tenth. The remaining 19 tags were readable after all 10 dry autoclave cycles. One tag failed after the first tissue processing cycle while the remaining 19 tags survived all 10 tissue processing cycles.

**Conclusions:**

In this preliminary study, these RFID tags show a high-degree of tolerance to tested solutions, solvents, temperature, and pressure conditions. However, a measurable failure rate is detectable under some circumstances and redundant identification systems such as barcodes may be required with the deployment of RFID systems. We have delineated testing protocols that may be used as a framework for preliminary assessments of candidate RFID tag tolerance to laboratory conditions.

## INTRODUCTION

Accurate and high-throughput identification is valuable for biospecimen tracking, particularly in anatomic pathology laboratories and pathology research laboratories. Mislabeling of blood samples and tissue specimens can lead to misdiagnosis for a patient[1] or potentially, in the case of research, misleading clinical trial results with potentially widespread impact. Radio frequency identification (RFID), a wireless data collection technology, is in the early phases of adoption in the pathology environment. The technology uses radio waves to remotely send and retrieve data contained in electronic tags, which are attached to or integrated into an object for the purpose of identification. This paper focuses on the tolerance of passive tags, which do not require an internal power source. Instead, radio frequency (RF) energy is absorbed by an RFID reader’s transmit function and converted into small amounts of electrical energy. The electrical energy powers the integrated circuit chip, which utilizes the remaining electrical energy to return its transmitted data payload to the reader’s receiver function, converting the tag’s signal into meaningful data strings. With no power source to wear down, passive RFID tags, when properly protected from environmental conditions, are known to survive for years and, in theory, can do so indefinitely while physically intact.

In the hospital setting, RFID-supported asset tracking has been used to significantly reduce errors in medical procedures, improve operating room efficiency, and increase patient-care time by freeing staff from clerical duties.[2] This asset tracking system has also allowed institutions to gain better inventory control and to plan for future investments.[3] In the pathology setting, RFID tags have been used to track specimen jars from endoscopy suites to the pathology accessioning area reducing labeling error rates from 9.29% (without RFID) to 0.55% (with RFID).[1] In addition, glass-encapsulated passive RFID tags placed in specimen cassettes have been used in a high-volume anatomic pathology lab to improve patient identification, drive workflow, and create an audit trail.[2,4] While bar codes generally require staff to scan assets manually, RFID tags potentially allow the scanning of cassettes and slides to be automatic. Integrating RFID tags into the cassettes and slides and building readers into workstations can increase the safety and reduce the probability of mistakes in histopathology processes.[2]

Unlike many other clinical settings where environmental conditions are relatively mild, the laboratory setting exposes RFID tags to harsh temperatures and solvents. In considering the use of RFID tags for anatomic pathology laboratories, there is little or no published data on tolerance of RFID tags to solvents and temperature extremes. Preliminary data was reported by M. Riben and colleagues reflecting some of the elements in this study.[5] For labeling glass slides, RFID tags must withstand high ambient temperatures such as those encountered during baking for deparaffinization or unrefrigerated transportation in the summer or through desert climes. Glass slides must also endure various solutions and solvents during hematoxylin and eosin (H&E) staining or immunostaining. With tissue cassettes, the RFID tag must resist solvents in the tissue processor. Cryovials or other containers may be subjected to autoclaving as well as ultralow temperatures in freezers or liquid nitrogen. An RFID tag may endure several cycles of freeze thawing when cryovials are removed and replaced. In this study, we evaluate readability of a candidate RFID tag after exposure to a range of solvent, temperature, and pressure conditions. As low costs of RFID tags depend on a high volume of production, it is preferable where possible to use one tag type to track a wide spectrum of laboratory objects rather than to develop and test a number of different RFID-tags for specialized uses. The Hitachi Mu-chip tag is a candidate for use in our laboratories because it does not require an internal power source and has a practical size for attachment to a variety of laboratory objects. The Hitachi Mu-chip is also designed specifically as an authentication/identification inclusion RFID device. Its unique identification number is structurally permanent within the chip and no two chips can contain the same ID number. We have, therefore, evaluated the robustness of the Hitachi Mu-chip tag across a spectrum of temperature, pressure, and solvent conditions that it might encounter and present a potential approach to testing of other types of tags.

## MATERIALS AND METHODS

Each Hitachi Mu-chip RFID tag consisted of a 0.4× 0.4× 0.12 mm thick integrated circuit Hitachi mu-chip (Hitachi America, Ltd., Brisbane, CA) attached to an aluminum 2.45 GHz 5.0 cm dipole antenna embedded in a pressure-sensitive adhesive sticker [Figure 1a]. The tags were attached to a tissue cassette, glass slide, or cryovial [Figures 1a, 1b, 2 and 3]. Tags used in H&E staining, and antigen retrieval studies were additionally completely covered by a layer of adhesive cryofilm (FPM200 – 2 mil white polyester/acrylic based adhesive/1.5 mil PET, CCL Label, Upland, CA), while tags in the tissue processing and high temperature and low temperature storage studies were not. For the autoclave studies, half the vials were covered by the additional film while the other half were not. The tags were interrogated by a Hitachi HSS-MUR-300 Reader (Hitachi America, Ltd.) at a distance of 1-2 inches from the reader. All tags were read once before experimental testing to obtain original 26-character identification numbers. This 26-character unique identification number is structurally hardwired into the RFID chip and essentially unalterable. Uniqueness of each identification number is maintained through a numbering protocol during manufacture where numbers are never duplicated or recycled. In addition, the chip and its reader use a proprietary communication protocol limiting the likelihood of ID (identification) collision with another vendor’s chip. A successful reading was scored when an RFID tag registered a unique 26-digit identification number that matched its original identification number and a nine-digit read event time stamp. An unsuccessful reading was marked when a tag did not register any identification number or time stamp whatsoever after five attempts at reading including reorientation of the tag.

**Figure 1a:**
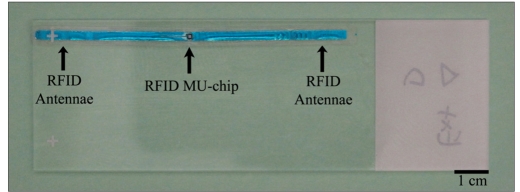
Slide Under-view. Viewed from below, the microchip is visible

**Figure 1b:**
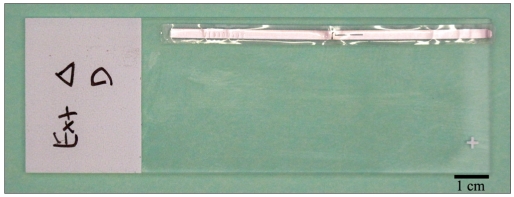
Slide top-view. The RFID tag and antenna were placed horizontally, without folding, along the length of the slide. While suitable for this study, the coverslip size would have to be reduced for actual use

**Figure 2:**
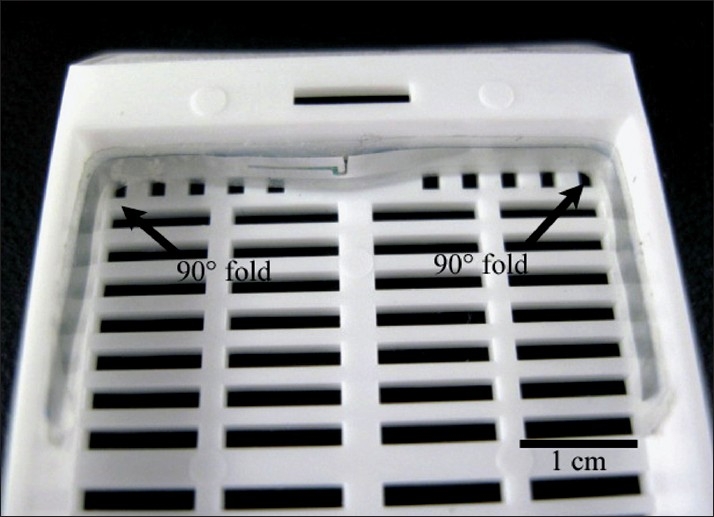
Tissue cassette. Given the length of the RFID antenna, folding to 90° in two places was required

**Figure 3:**
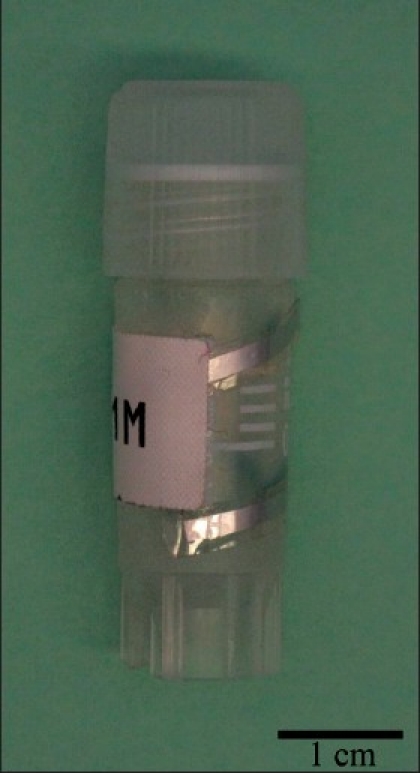
Cryovial. RFID tags were wrapped around the cryovial in a spiral, while tag labels were placed over the tags in the middle section of the cryovial

### Autoclaving

As autoclaving may be used to sterilize containers in the laboratory, we evaluated tag durability in these high temperature and pressure conditions. RFID tags were attached to 40 empty 1-ml capacity cryovials (Corning Inc., Corning, NY). Twenty vials were autoclaved using the dry cycle setting of a Steris Amsco Century SV-120 autoclave (Steris Corp., Mentor, OH) and allowed to cool to room temperature before reading of the RFID tag. Ten of these dry autoclave vials were covered entirely by cryofilm, while another 10 vials were not. The dry autoclave cycle consisted of sterilization at 121°C and 26 pounds per square inch (psi) for 20 minutes followed by drying for 20 minutes at atmospheric pressure (15 psi).

These vials were autoclaved 10 times and read after each autoclaving. Another 20 RFID-labeled cryovials were autoclaved using the liquid or “wet” cycle settings. Ten of these liquid autoclave vials were covered entirely by cryofilm, while another 10 vials were not. The liquid cycle consisted of sterilization for 20 minutes at 15 psi and 121°C, differing from the dry cycle in that it lacked an extra drying phase and used lower sterilization pressure.[6] These vials were autoclaved 10 times and read after each cycle as well.

### Tissue Processing

After fixation of tissue and before embedding, plastic cassettes containing tissue are commonly run through a tissue processor. To replicate this process, 20 RFID tags were each affixed to the inside of a tissue processing cassette. Owing to its length, the RFID antennae of each tag were folded to 90° in two places to fit the tissue cassettes [Figure 2]. Initial RFID readings were obtained before enduring 10 complete cycles of tissue processing on a tissue processor (Sakura Tissue-Tek VIP, Sakura Finetek USA, Torrance, CA). Each cycle consisted of the following steps: 1) treatment in 80% ethanol for 105 minutes, 2) three treatments of 95% ethanol for 45 minutes, 105 minutes, and 45 minutes, 3) three treatments of 100% ethanol for 105 minutes, 45 minutes, and 45 minutes, 4) three treatments of xylene for 105 minutes, 45 minutes, and 45 minutes, 5) four treatments of paraffin for 105 minutes, 45 minutes, 45 minutes, and 45 minutes, and 6) a cleaning cycle consisting of a xylene treatment followed by an alcohol treatment. The ethanol and xylene treatments were performed at 40°C and the paraffin treatment at 60°C. After each complete cycle, an RFID reading was obtained for each cassette.

### Hematoxylin and Eosin Staining

Twenty blank charged glass slides were each affixed with an RFID tag. Each tag on the slide was entirely covered with a 20×60 mm piece of cryofilm before obtaining original RFID readings and 10 consecutive cycles of H&E staining. Tags were placed horizontally along the length of the slides [Figures 1a and 1b]. The protocol for each H&E staining run consisted of immersions in the following solvents/dyes: 1) xylene (15 minutes), 2) graded alcohols (100, 95, 80, and 70% ethanol, 2 minutes each), 3) distilled water (2 minutes), hematoxylin (2 minutes), 4) cold water rinse until excess hematoxylin was washed off the slides, 5) eosin (3 minutes), graded alcohols (70, 80, 95, and 100%, 2 minutes each), and 5) xylene (15 minutes). Each slide was subjected to 10 consecutive staining runs. An RFID reading was obtained for each slide after each H&E run. It should be noted that the solutions and solvents often loosened the tags requiring reapplication.

### Pressure Cooker for Antigen Retrieval

Twenty RFID-tagged glass slides were placed in a solution of citrate buffer (pH=6) in a Biocare Decloaking Chamber (Biocare Medical, Concord, CA) for 30 minutes. Each RFID tag was secured and covered completely with cryofilm. The slides were heated to a maximum temperature of 125°C for 5-10 minutes at 15 psi and cooled to 90°C before removal from the pressure cooker (total time of run=30 minutes). The slides were cooled with three changes of room temperature (22°C) tap water for 10 minutes. A KJT-Thermocouple Thermometer (Hanna Instruments, Woonsocket, RI) was used to confirm cooling to room temperature. An RFID reading was obtained for each slide afterwards. This process was performed a total of 10 times.

### High Temperature Conditions (52 ֿ75°C for 7 days)

Glass slides may be baked in a 60°C oven for 12-18 hours prepatory to immunostaining. In addition, ambient temperatures during the summer or in the desert may approach 50 °C with intravehicular temperatures reaching up to 75°C during the day.[7] During the evening, desert ambient and intravehicular temperatures are substantially lower, typically around 30°C and 52°C, respectively.[8,9] Such conditions are of particular concern in Southwestern states such as California, Arizona, and Nevada.

To simulate desert day-night cycles over 7 days, 20 blank charged slides were tagged and placed in a 75°C oven (Biocare Desert Chamber Dry2008US, Biocare Medical, Concord, CA) for 8 hours during daytime hours and in a separate 52°C oven (Yamato Constant Temperature Oven DKN402, Yamato Scientific Co., Ltd., Tomioka, Koto-ku, Tokyo) for 16 hours overnight. An RFID reading of each slide was obtained during each oven transfer for a total of two readings per slide per day (280 readings total). This procedure was performed every day for 7 consecutive days and 7 consecutive nights.

To test tolerance for sustained high temperatures, 20 blank charged slides were tagged and placed in the 75°C oven for an entire week. RFID readings of the tags were obtained twice daily (at 9 am and at 5 pm) immediately upon removal of the slides from the oven.

### Cold Temperature Conditions (-80°C, -150°C, -196°C up to 12 months)

Biospecimens are commonly stored at extreme cold temperatures in electrical freezers (-80°C, -150°C) or sometimes in liquid-phase liquid nitrogen (-196°C). In addition, these specimens often undergo freeze-thawing multiple times when they are retrieved for research.

Thirty RFID-tagged 1 ml cryovials each containing 0.15-0.3g of fresh autopsy brain tissue and another 30 cryovials each containing 1 ml of cell-culture media (ATCC, DMEM/F12, 1:1; Manassas, VA) were stored for 3 months at selected low temperatures. Three separate groups of 10 tissue-containing vials and 10 liquid-filled vials were placed in: 1) the liquid phase of a -196°C liquid nitrogen tank (Thermolyne Locator 4 FFF Cryo Biological Storage System, Asheville, NC), 2) a -150°C freezer (Sanyo VIP Plus -150°C Cryogenic Freezer MDF-C2156VANC, Sanyo North America, San Diego, CA), and 3) a -80°C freezer (Thermo Scientific Revco Elite Plus, ThermoFisher Scientific, Waltham, MA). Original RFID readings were obtained for each group of vials before storage in their respective cryogenic conditions. After 3 months, the vials were thawed for 3 minutes in a 37°C warm water bath (ThermoScientific, Waltham, AL). RFID readings were obtained when room temperature (22°C) was attained. A KJT-Thermocouple Thermometer (Hanna Instruments, Woonsocket, RI) was used to confirm warming to room temperature. After being retrieved and interrogated for the 3-month timepoint, the same 30 cryovials containing fresh autopsy brain tissue and second 30 cryovials containing cell-culture media were returned to their respective freezers and stored for another 9 months. Readings were taken as before and recorded at 12 months.

### Fifty Freeze-thaw Cycles (-196°C to 22°C)

As RFID tag components may potentially be affected by contraction and expansion effects during freezing and thawing, we evaluated the freeze-thawing of tags. Ten RFID-tagged vials containing fresh autopsy brain tissue (0.15-0.30 g per vial) and 10 vials containing cell-culture media (1.0 ml per vial) were submerged in liquid-phase liquid nitrogen until their internal temperatures were stable at -196°C. Original RFID readings were obtained before submerging the vials into the liquid nitrogen. Approximately 4 minutes were required for all tags to reach -196°C. A KJT-Thermocouple Thermometer (Hanna Instruments, Woonsocket, RI) was used to confirm the temperature. The vials were then thawed for 3 minutes in a warm water bath (37°C) to 22°C. An RFID reading was obtained after each freeze-thaw cycle. This process was performed 50 times for each cryovial.

## RESULTS

Glass microscopic slides and formalin-fixed paraffin-embedded (FFPE) tissue blocks may be subjected to high temperatures during storage or transportation. We have observed melted paraffin blocks related to air-conditioning failures during summer or during transportation through desert or semi-desert regions (e.g. Nevada) suggesting intravehicular temperatures exceeding 58°C, a typical melting point for paraffin compounds. In our laboratory, we typically deparaffinize slides in a 60°C oven for 1-18 hours. During summer and in desert conditions common to parts of the Southwest United States, ambient temperatures are known to reach 50°C with intravehicular temperatures capable of approaching 75 °C.[7] We therefore chose 75°C as an approximation of a high temperature condition. The Hitachi Mu tags functioned well under these conditions for a week as well as temperature changes that mimicked day-night cycles [Table 1].

**Table 1:** High temperature conditions

Condition tested	Number of tags	Successful reads	Failed read
Dry autoclave 121oC/26psi 10×	20	199	1 tag, 10^th^ cycle (5% failure rate; 1 of 20 tags)
Wet autoclave 121oC/15 psi 10×	20	200	None
75°C/52°C, 7 days	20	280	None
75°C, 7 days	20	140	None

Autoclaving is a common means of sterilizing containers or equipment in laboratories. This use might be encountered most often in a pathology research laboratory. Autoclaving is a convenient way to provide a brutal test of survivability in the face of elevated temperature, pressure, and moisture. The vast majority of the RFID tags (39 of 40) did survive autoclaving where temperatures reach 121°C, providing further evidence of tolerance to high temperatures. The solitary tag that failed was readable after nine full autoclave cycles but not after the tenth [Table 1]. It is unclear, however, whether that failure was due to temperature, pressure, moisture, or some other factor. The failed tag (1 of 10 uncovered tags) was not covered by cryofilm suggesting that even the modest shielding provided by a layer of cryofilm may be beneficial. In contrast, none of the 10 covered RFID tags failed.

At the other end of the spectrum, pathologists commonly freeze tissues for tumor or tissue banks. The RFID tags performed well with 60 tags functional after 3 months and 12 months at temperatures ranging from -80°C to -196°C [Table 2]. As impressively, 20 individual tags subjected to a cumulative 1000 freeze-thaw events in liquid nitrogen were interrogated successfully after each cycle. In our experience, cryovials are generally frozen and thawed a handful of times so the survival of each RFID tags after 50 freeze-thaw events is encouraging. In summary, these passive RFID tags perform robustly at the low or high extremes of temperature that might be encountered in our pathology laboratory settings.

**Table 2:** Freezing temperature conditions

Condition tested	Number of tags	Successful reads	Failed read
-196°C/22°C, 50×	20	1000	None
-80°C, 12 months	20	40 (at 3, 12 months)	None
-150°C, 12 months	20	40 (at 3, 12 months)	None
-196°C, 12 months	20	40 (at 3, 12 months)	None

Tissue processing, H&E staining, as well as immunostaining are common procedures in the pathology laboratory. These processes may require immersion of devices in water-based solutions or solvents (ethanol and xylene). RFID tags attached to slides were given modest shielding from H&E solutions through the adhesive acrylic film covering. As the solvents tended to dissolve film adhesive and consequently loosen or remove film covering, the film required reattachment on multiple occasions. Affected tags functioned well despite lapses in protective covering [Table 3].

**Table 3:** Laboratory procedures

Condition tested	Number of tags	Successful reads	Failed read
Tissue processing 10×	20	190	1 tag, 1st cycle (5% failure rate; 1 of 20 tags)
H & E staining 10×	20	200	None
Pressure cooker antigen retrieval 10×	20	200	None

For RFID tags applied to tissue cassettes and subjected to tissue processing, one tag failure occurred immediately after one cycle [Table 3]. The remaining 19 tags, however, survived 10 cycles of tissue processing, suggesting a relatively high tag tolerance to solutions and solvents in the processor. It is unclear as to why there was one case of near instant failure. We have noted anecdotally that physical manipulation of RFID tags alone can cause sufficient damage to preclude interrogation. For the tissue cassettes, the RFID antennae were folded to 90o in two places to fit the tissue cassettes [Figure 2]. This manipulation may have increased the likelihood of tag failure. Physical damage in combination with solutions and solvents at elevated temperatures is suspected. We cannot completely exclude the possibility of a contributory manufacturing defect.

One limitation of RFID tags is that their metallic content contraindicates the use of a microwave for antigen retrieval before immunostaining. Another method of antigen retrieval is the use of a pressure cooker. Exposure to 125°C at 15 psi for 30 minutes in the tested pressure cooker was well-tolerated with no failures despite 10 cycles of testing. We did not subject RFID tags to immunostaining itself as solutions and solvents (water, xylene, alcohol) are similar to those of H&E staining and tissue processing.

## CONCLUSIONS

In this limited and preliminary study, RFID tags show a level of tolerance to extremes of temperature and solvent conditions. We demonstrate that repeated freeze-thaw cycles as well as extended storage for up to 1 year at low temperatures are well tolerated. We have presented a variety of conditions under which other RFID tags may be tested before introduction into use in pathology laboratories. Such a small, preliminary study might be useful as a prelude to a larger scale (and more costly) testing regime. Rare failures do occur and these are of concern in that the number of fully deployed RFID tags for a pathology information system may be in the hundreds of thousands to millions. Even an ostensibly “small” failure rate of 0.1% in 1,000,000 tags, means that 1,000 samples are not readable while a 5% failure rate would equate to 50,000 unreadable tags. A level of redundancy is then desirable; either a barcode and/or legible information is likely necessary. Some observations were made indirectly related to the intent of the original study. If a single type of RFID tag is to be deployed for multiple applications there are 3 additional considerations: shielding, size, and mode of attachment. From a practical viewpoint, it was challenging to apply the RFID tag given the length of the antenna to slides and cassettes so the shape and profile of a tag will be important. As physical manipulation was often required with the current tag, RFID tags may need to be shortened or resized to fit slides and cassettes. As is, our current mode of attachment to glass slides reduces the area for placement of tissue sections and cover slips. Shielding from solutions and solvents may be desirable as direct exposure to these solvents may contribute to tag failure, although, in a surprising minority of cases. If shielding should fail, our data suggests that at least this Hitachi tag is likely to survive a majority of environmental insults. Our mode of attachment which was essentially an adhesive sticker or film did not tolerate solvents well. Therefore, alternative means of attachment or integration into the object is desirable. Varying modes of RFID integration into tracked objects or different types of RFID tags may be necessary potentially raising the cost of using such tags.
